# SERCA2a gene transfer improves electrocardiographic performance in aged mdx mice

**DOI:** 10.1186/1479-5876-9-132

**Published:** 2011-08-11

**Authors:** Jin-Hong Shin, Brian Bostick, Yongping Yue, Roger Hajjar, Dongsheng Duan

**Affiliations:** 1 Department of Molecular Microbiology and Immunology, School of Medicine, The University of Missouri, Columbia, MO, USA; 2 Department of Cardiology, Cardiovascular Research Center, Mount Sinai School of Medicine, New York, NY, USA

## Abstract

**Background:**

Cardiomyocyte calcium overloading has been implicated in the pathogenesis of Duchenne muscular dystrophy (DMD) heart disease. The cardiac isoform of sarcoplasmic reticulum calcium ATPase (SERCA2a) plays a major role in removing cytosolic calcium during heart muscle relaxation. Here, we tested the hypothesis that SERCA2a over-expression may mitigate electrocardiography (ECG) abnormalities in old female mdx mice, a murine model of DMD cardiomyopathy.

**Methods:**

1 × 10^12 ^viral genome particles/mouse of adeno-associated virus serotype-9 (AAV-9) SERCA2a vector was delivered to 12-m-old female mdx mice (N = 5) via a single bolus tail vein injection. AAV transduction and the ECG profile were examined eight months later.

**Results:**

The vector genome was detected in the hearts of all AAV-injected mdx mice. Immunofluorescence staining and western blot confirmed SERCA2a over-expression in the mdx heart. Untreated mdx mice showed characteristic tachycardia, PR interval reduction and QT interval prolongation. AAV-9 SERCA2a treatment corrected these ECG abnormalities.

**Conclusions:**

Our results suggest that AAV SERCA2a therapy may hold great promise in treating dystrophin-deficient heart disease.

## Background

The heart is often afflicted in Duchenne muscular dystrophy (DMD), a lethal muscle disease caused by dystrophin deficiency (reviewed in [1]). Dystrophin is a large sub-sarcolemmal protein that plays a critical role in maintaining sarcolemma integrity. In a dystrophin-deficient heart, myocardial contraction results in sarcolemmal damage. Subsequent cardiomyocyte necrosis and fibrosis leads to dilated cardiomyopathy. The exact molecular mechanisms underlying dystrophin-deficient heart disease remain to be fully clarified. Interestingly, ample evidence suggests that abnormal elevation of cytosolic calcium may play a central role in the pathogenesis of DMD heart disease [2-6].

The sarcoplasmic reticulum is the primary calcium storage organelle in muscle cells. In cardiomyocytes, removal of cytosolic calcium is mainly accomplished by the cardiac isoform of sarcoplasmic reticulum calcium ATPase (SERCA2a) via its pump activity (reviewed in [7]). Basically, SERCA2a actively transports calcium from the cytosol to the sarcoplasmic reticulum during myocardial relaxation. SERCA2a expression/activity is reduced in various forms of heart failure in experimental animal models and human patients (reviewed in [8,9]). In the heart of dystrophin-deficient mdx mice, SERCA2a expression is also significantly decreased [10]. Here, we hypothesize that intentional SERCA2a over-expression may help mitigate cytosolic calcium overload and improve cardiac electrophysiology in symptomatic mdx mice.

Among various gene transfer vectors, adeno-associated virus serotype-9 (AAV-9) is by far the most robust vector for transducing the mdx heart when administrated intravascularly [11-13]. We have recently established the aged female mdx mice as an authentic model of DMD cardiomyopathy [14,15]. To test our hypothesis, we delivered 1 × 10^12 ^viral genome (vg) particles/mouse of AAV-9 SERCA2a vector to 12-m-old female mdx mice via a single bolus tail vein injection. Electrocardiography (ECG) was performed when mice reached 20 months of age. Compared to that of age- and gender-matched untreated mdx mice, the ECG profile of AAV-9 SERCA2a treated mdx mice was significantly improved.

## Methods

### Recombinant AAV-9 SERCA2a vector

**Figure 1:**
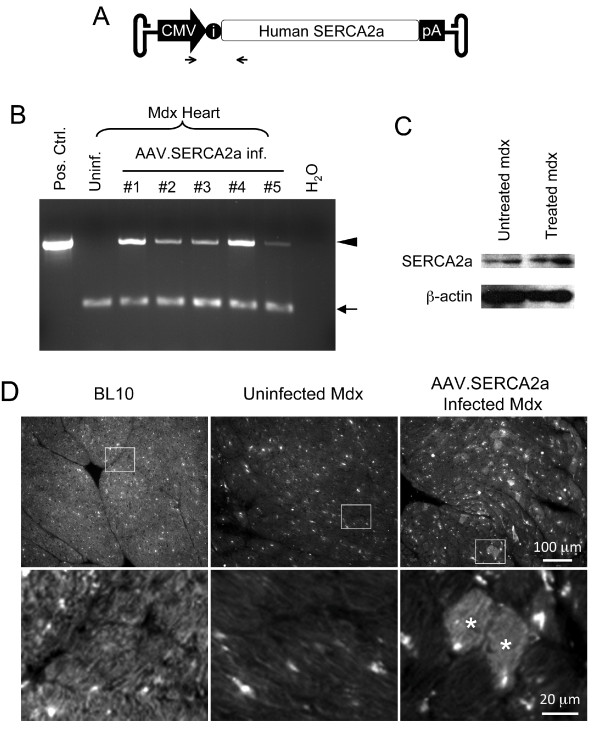
**AAV-9 mediated SERCA2a transduction in the mdx heart**. **A**, Schematic outline of the AAV SERCA2a vector used in the study. The human SERCA2a cDNA is driven by the CMV promoter. i, intron. Arrows mark the locations of the PCR primers. **B**, PCR detection of the AAV SERCA2a vector genome in the mdx heart. Pos. Ctrl., the SERCA2a *cis *plasmid; Uninf., from an uninfected mdx heart; #1 to #5, from five AAV-9 SERCA2a vector infected mdx mouse hearts. Each line represents PCR result from one mouse; H_2_O, no DNA was added in the PCR reaction. Arrowhead, the 519 bp diagnostic band for the AAV SERCA2a genome; Arrow, the 160 bp diagnostic band for the CFTR gene (internal control). **C**, Representative SERCA2a western blot. β-actin was used as the loading control. **D**, Representative SERCA2a immunofluorescence staining images from BL10, mdx and AAV-9 SERCA2a infected mdx hearts. Enlarged images (bottom panels) are the boxed areas from the corresponding low-power photomicrographs (top panels). Asterisk, AAV SERCA2a transduced cardiomyocytes.

The *cis *plasmid for AAV-9 SERCA2a vector production has been extensively characterized and used in various animal studies and human trials [16-19]. In this construct, the human SERCA2a cDNA expression was regulated by the ubiquitous cytomegalor virus (CMV) promoter, a hybrid intron and a bovine growth hormone poly-adenylation signal (Figure 1A). Experimental AAV vector was produced using a previously reported triple plasmid transfection protocol [20,21]. Recombinant viral stocks were purified through two rounds of isopycnic CsCl ultracentrifugation as we previously described [22]. Viral titration and quality control were performed according to our published protocol [22,23].

### In vivo gene delivery

All animal experiments were approved by the Animal Care and Use Committee of the University of Missouri and were in accordance with NIH guidelines. Dystrophin-deficient mdx mice and normal control C57Bl/10 (BL10) mice were purchased from The Jackson Laboratory (Bar Harbor, ME). AAV-9 SERCA2a vector was injected to conscious 12-m-old mdx mice in a single bolus through the tail vein according to a previously described protocol [11]. Each mouse received 1 × 10^12 ^vg particles of AAV-9.

### PCR detection of the AAV vector genome

DNA was extracted from frozen heart tissue sections as we described before [24]. The AAV SERCA2a vector genome was amplified with a forward primer corresponding to the CMV promoter (DL1263, 5'-CCAAGTACGCCCCCTATTGA) and a reverse primer corresponding to the human SERCA2a cDNA (DL1262, 5'- AGCCCCGTACTCTCGTTGAC) (Figure 1A). The size of the expected PCR product is 519 bp. The mouse CFTR gene was used as an internal control. The forward primer corresponds to the mouse cystic fibrosis transmembrane conductance regulator (CFTR) gene exon 2 (DL1286, 5'-CATATACCAAGCCCCTTCTGCT). The reverse primer corresponds to the mouse CFTR gene intron 2 (DL1287, 5'- TGCATCACTTTTAAATGGAACCTC). The expected mouse CFTR gene amplicon size is 160 bp.

### Western blot

The frozen heart was ground to fine powder in liquid nitrogen. Whole heart muscle lysate was prepared according to our published protocol [15,25]. Primary antibody of for SERCA2a (1:3,000) has been previously described [26]. A monoclonal antibody to β-actin (1:5,000, Sigma; St Louis, MO) was used to confirm protein loading.

### SERCA2a immunofluorescence staining

SERCA2a expression was confirmed by immunofluorescence staining. Briefly, 10 μm frozen heart sections was blocked with 20% goat serum at room temperature for 30 min. The rabbit polyclonal anti-SERCA2a antibody was then applied at the dilution of 1:3,000 overnight at 4°C [26]. SERCA2a staining was revealed with an Alex 488 conjugated goat anti-rabbit antibody (1:100 dilution).

### Histopathology examination

General heart histology was evaluated by hematoxylin and eosin (HE) staining. Cardiac fibrosis was examined by Masson trichrome staining as we described before [27]. Fibrotic tissue stained blue and myocardium stained dark red.

### ECG examination

Mice were anesthetized with isoflurane (3% induction, 1-1.5% maintenance). A non-invasive 12-lead ECG was performed according to our published protocol [28]. ECG signals were processed through a single channel bioamplifier (Model ML132; AD Instruments) and then recorded on a Model MLA0112S PowerLab system using the Chart software (version 5.5.5, AD Instruments, Colorado Springs, CO). ECG from a continuous 1 min recording was analyzed by the Chart ECG analysis software (version 2.0, AD Instruments). The amplitude of the Q wave was analyzed using the lead I tracing. The remaining ECG parameters were analyzed using lead II tracing results. Cardiomyopathy index is determined by dividing the QT interval with the PQ segment (QT/PQ).

### Statistical Analysis

Data are presented as mean ± standard error of mean. Statistical analysis was performed with the SPSS software (SPSS, Chicago, IL) using one-way ANOVA followed by Bonferroni *post hoc *analysis. Difference was considered significant when *P *< 0.05.

## Results

### AAV-9 mediated SERCA2a gene transfer in old mdx mice

To evaluate SERCA2a gene therapy in a dystrophin-deficient heart, we packaged the CMV.SERCA2a construct into AAV-9 (Figure 1A). Since the heart of young mdx mice is mildly affected, we opted to test SERCA2a therapy in 12-month-old mdx mice [29]. At this age, mdx mice exhibit cardiac histopathology but do not suffer heart failure [29]. The CMV.SERCA2a vector has been extensively characterized in different animal models and is currently in use in a human trial [17-19,30,31]. We injected AAV-9 SERCA2a to 12-m-old mdx mice via the tail vein. Eight months later, we examined the AAV genome in the heart. The vector genome was detected in all mdx mice that received AAV-9 SERCA2a injection but not in untreated mdx mice (Figure 1B). To confirm SERCA2a expression, we performed western blot and immunofluorescence staining. Compared with untreated mdx, increased SERCA2a expression was found in AAV infected mdx mice by western blot (Figure 1C). Consistent with previous reports [10,32], we observed endogenous cytosolic SERCA2a staining in the BL10 heart by immunostaining (Figure 1D). Further, the endogenous SERCA2a level was reduced in the mdx heart (Figure 1D). Consistent with the published AAV-9 transduction profile in the mdx heart [11,12], we observed mosaic but widespread AAV-mediated SERCA2a expression in the hearts of AAV-9 SERCA2a infected mdx mice (Figure 1D).

### AAV-9 SERCA2a therapy improved ECG performance

**Figure 2:**
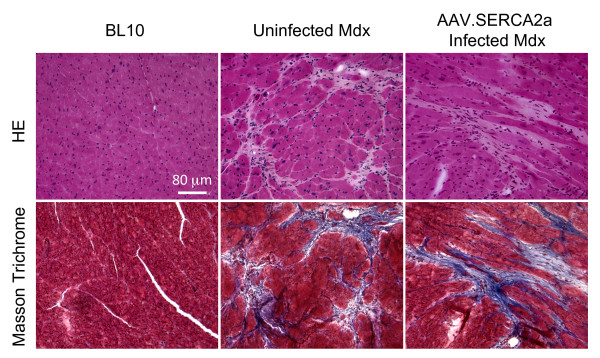
**SERCA2a expression does not mitigate histological lesions in the mdx heart**. Top panels, representative HE staining images; Bottom panels, representative Masson trichrome staining images.

**Figure 3:**
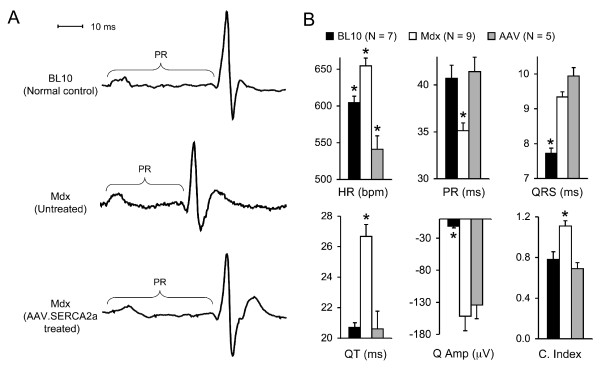
**AAV-9 SERCA2a expression improves the ECG profile in mdx mice**. **A**, Representative single lead II tracings from BL10, mdx and AAV SERCA2a treated mdx mice. PR, the time interval between the onset of atrial depolarization and the onset of ventricular depolarization. **B**, Quantitative evaluation of ECG profiles in BL10, mdx, AAV SERCA2a treated mdx mice. _*_, Statistically different from other groups. HR, heart rate; PR, PR interval; QRS, QRS duration; QT, QT interval; Q Amp, Q amplitude in lead I; C. Index, cardiomyopathy index.

On histopathologic examination, the hearts of SERCA2a treated mice were not different from those of untreated mdx mice (Figure 2). Myocardial fibrosis was clearly observed in the hearts of both treated and untreated mdx mice (Figure 2). Surprisingly, ECG examination revealed significant improvement (Figure 3). Specifically, tachycardia was corrected. The PR interval, QT interval and cardiomyopathy index were normalized (Figure 3B). Interestingly, the widened QRS duration and the deep Q wave were not improved (Figure 3B).

## Discussion

Cardiac complications are a major health issue in DMD. Current treatments are limited to symptomatic medications and heart transplantation [33]. In an effort to develop more effective therapies, several experimental gene therapy approaches have been explored in the rodent models [29]. These include AAV-mediated expression of an abbreviated synthetic dystrophin gene and antisense oligonucleotides-mediated exon skipping [12,13,34-36]. In general, the goal of these strategies is to express a truncated yet functional dystrophin protein. While these attempts are highly encouraging, a recent clinical trial suggests that immunity to dystrophin may represent a significant barrier [37]. Alternative strategies based on endogenous genes may offer immune advantages compared to dystrophin replacement/repair therapies.

Over the last decade tremendous progress has been made in our understanding of the pathogenesis of DMD cardiomyopathy. An emerging theme is the disruption of calcium homeostasis (reviewed in [38,39]). First, stress-induced calcium influx is significantly increased in mdx cardiomyocytes. Extracellular calcium may enter through stretch-activated calcium channel (such as TRPC1), sarcolemmal microrupture and sodium-calcium exchanger [4,40,41]. Second, calcium may leak from the sarcoplasmic reticulum via phosphorylated and/or S-nitrosylated ryanodine receptor 2 [5,6]. Collectively, these studies suggest that calcium overloading may represent a major pathogenic mechanism in DMD heart disease. Since SERCA2a plays a major role in calcium removal in the heart, we reasoned that forced expression of SERCA2a via AAV gene transfer might benefit dystrophin-deficient heart. We observed AAV genome persistence and SERCA2a over-expression in the hearts of 20-m-old mdx mice that were treated at age of 12 months (Figure 1). In support of our hypothesis, the ECG profile was significantly improved in AAV SERCA2a treated mice (Figure 3).

AAV SERCA2a therapy has successfully reversed cardiac dysfunction in several large animal models [17,30]. A Phase I trial has revealed an excellent safety profile [18,19]. Recently released results from the Phase II trail have further established clinical efficacy of AAV SERCA2a therapy in treating advanced heart failure [31]. While additional in vitro analysis of myocardial contractility and in vivo evaluation of hemodynamics (echocardiography and cardiac catheter) are needed [42], our results demonstrate for the first time that AAV SERCA2a may hold great promise in alleviating cardiac disease in DMD patients. Consistent with our findings in the heart, a recent study suggests that AAV SERCA2a also significantly reduced skeletal muscle disease in dystrophic mice following local gene transfer [43].

## Conclusions

Our results here have opened a new avenue to treat DMD cardiomyopathy using AAV SERCA2a gene delivery. Future studies in aged mdx mice, dystrophin/utrophin double knockout mice and dystrophin-deficient dogs may further validate AAV SERCA2a mediated gene therapy for DMD.

## List of abbreviations

AAV: adeno-associated virus; BL10: C57Bl/10; CFTR: cystic fibrosis transmembrane conductance regulator; CMV: cytomegalovirus; DMD: Duchenne muscular dystrophy; ECG: electrocardiography; HE: hematoxylin and eosin; PCR: polymerase chain reaction; SERCA2: cardiac isoform of sarcoplasmic reticulum calcium ATPase; vg: viral genome.

## Competing interests

Dr. Hajjar has ownership interest (include stock options and rights in patents) in Celladon Corporation, a company involved in SERCA2a clinical trials. The other authors declare that they have no competing interest.

## Authors' contributions

BB participated in ECG assay. DD conceived of study and wrote the manuscript. JS performed PCR, western blot, immunostaining, histology and ECG assay. RH provided critical reagents and advice. YY made AAV vector and participated in morphology and ECG studies. All authors read and approved the final manuscript.
